# Pulmonary Tumor Thrombotic Microangiopathy from Metastatic Prostate Carcinoma

**DOI:** 10.1155/2015/286962

**Published:** 2015-01-06

**Authors:** Dhruv Nayyar, Kavitha Muthiah, Christopher S. Hayward, Zerlene Lim, Emily K. Granger, Mark Nicholls, Allan R. Glanville

**Affiliations:** St. Vincent's Hospital, Victoria Street, Darlinghurst, Sydney, NSW 2010, Australia

## Abstract

Pulmonary tumor thrombotic microangiopathy is a rare but serious malignancy-related respiratory complication. The most common causative neoplasm is gastric adenocarcinoma. We report a case caused by metastatic prostate adenocarcinoma, diagnosed postmortem in a 58-year-old male. To our knowledge, this is the second reported case from metastatic prostate adenocarcinoma.

## 1. Introduction 

Pulmonary tumor thrombotic microangiopathy (PTTM) is an underrecognized and aggressive condition. It should be considered in the differential diagnosis of pulmonary hypertension, unresponsive to conventional therapy, particularly in patients with known cancer. Antemortem diagnosis may be facilitated through judicious use of pulmonary microvascular cytology, imaging modalities, and lung biopsy. We report the second case of PTTM from metastatic prostate carcinoma.

## 2. Case Presentation

A 58-year-old male presented with a two-week history of progressive dyspnea on exertion with no associated symptoms. He had hypertension and was a heavy smoker but had no history of malignancy. On examination, he was afebrile, normotensive, tachycardic (108 beats per minute), and tachypneic (28 breaths per minute). Peripheral oxygen saturation was 70% on room air. He displayed increased work of breathing. There were no other abnormal examination findings.

**Figure 1:**
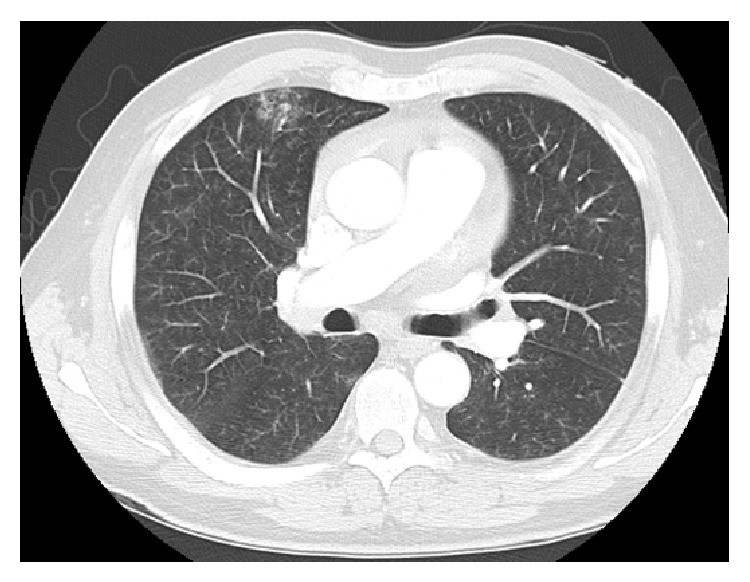
CTPA indicating subtle bilateral and diffuse bronchiolar densities with tree-in-bud appearance but no evidence of pulmonary embolism.

Full blood count and blood chemistry were normal. Arterial blood gas measurement revealed hypoxemia with chronic respiratory alkalosis (pH 7.45,* p*O_2_ 37 mmHg,* p*CO_2_ 22 mmHg). Electrocardiogram showed sinus tachycardia. Chest radiograph indicated clear lung fields and mild cardiomegaly. Transthoracic echocardiogram revealed severe pulmonary hypertension (pulmonary artery pressure 90 mmHg) and right ventricular dilatation with impaired right ventricular systolic function. Left ventricular size and systolic function were normal. Computed tomography pulmonary angiogram (CTPA) revealed subtle bilateral and diffuse bronchiolar densities with tree-in-bud appearance but no evidence of pulmonary embolism (Figure 1). A ventilation-perfusion (V/Q) scan was not performed. Pulmonary function testing was normal.

The working diagnosis at this stage was pulmonary hypertension with the cause being not yet determined (severe hypoxemia suggested that idiopathic pulmonary hypertension was unlikely). He was commenced on iloprost but was not responding so epoprostenol was subsequently added. Despite the institution of treatment, his pulmonary pressures remained elevated.

The patient's condition continued to deteriorate in the subsequent three days with worsening respiratory failure. He was transferred to the intensive care unit, intubated, and commenced on venoarterial extracorporeal membrane oxygenation (VA-ECMO), potentially as a bridge to lung transplantation. Invasive diagnostic procedures including right heart catheterization and lung biopsy were considered to have an unfavorable risk-benefit ratio. He developed right-sided heart failure with pedal edema and an elevated jugular venous pressure. Chest radiograph showed bilateral patchy infiltrates. Bronchoscopy revealed brown fluid consistent with alveolar hemorrhage.

**Figure 2:**
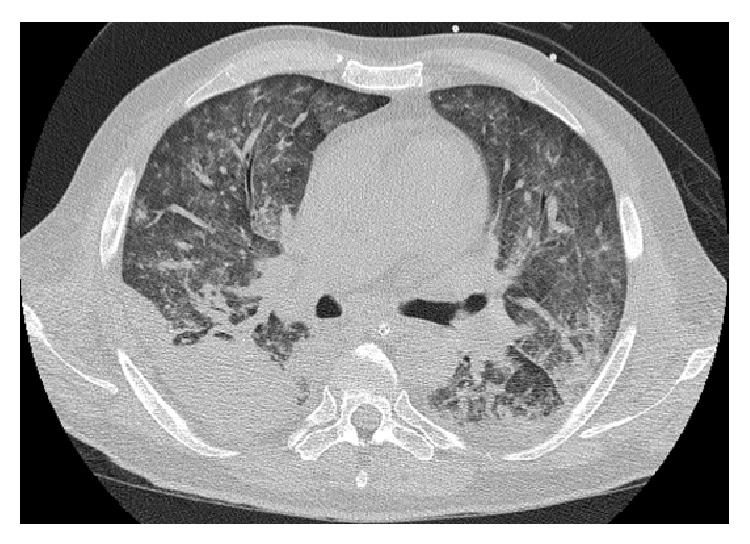
Chest CT scan demonstrating diffuse ground glass opacification with consolidation and pleural effusion. On autopsy, the lungs showed evidence of haemorrhagic infarction in keeping with the CT appearance.

Persistent hematuria from the time of urinary catheterization suggested a possible urological problem. Prostate specific antigen was markedly raised at 109 ug/L. A CT scan showed multiple sclerotic and lytic lesions in the lower thoracic and lumbosacral spine and pelvis, suggestive of metastatic prostatic carcinoma. There was diffuse ground glass opacification with consolidation and pleural effusion in the lungs (Figure 2). After full discussion with family members, invasive ventilatory support was withdrawn, palliative treatment was commenced, and the patient died shortly thereafter due to respiratory failure.

**Figure 3:**
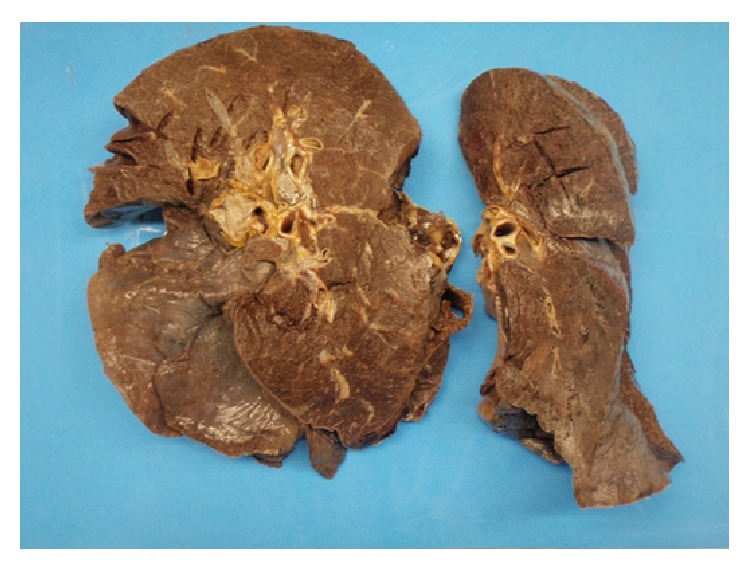
Lung cut surfaces showing bilateral heavy and hemorrhagic lungs. The left lung weighed 1050 grams and the right weighed 1350 grams. Both lungs were macroscopically hemorrhagic which was confirmed on microscopy to be due to hemorrhagic infarction.

**Figure 4:**
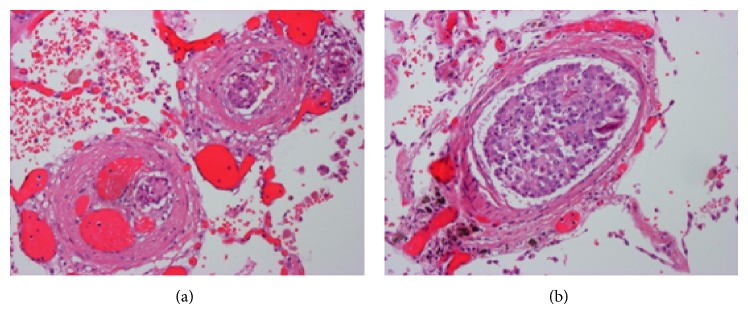
Lung tissue showing small and medium sized arteries with intimal thickening and luminal narrowing and recanalization, with tumor emboli present (hematoxylin-eosin, original magnification ×200).

At autopsy, the patient was found to have adenocarcinoma of the prostate (Gleason score 8) with multiple metastatic tumor emboli within the lungs, adrenal glands, bladder, thyroid, and lumbar vertebrae. Macroscopic findings included bilateral heavy and hemorrhagic lungs (Figure 3). Histology revealed features of PTTM in small and medium sized arteries within both lungs including intimal thickening, luminal narrowing and recanalization, and tumour emboli (Figure 4). Vascular changes consistent with pulmonary hypertension were also present.

## 3. Discussion

The most frequent causative neoplasm for PTTM is poorly differentiated gastric adenocarcinoma. Other causes include gastric signet ring cell carcinoma, gallbladder carcinoma, and adenocarcinomas of the pancreas, breast, liver, colon, lung, and ovary [1]. There has been one other reported case of PTTM caused by prostate adenocarcinoma [1].

In PTTM, tumour cells metastasize to the pulmonary arterial system and adhere to the vascular endothelium [2]. This activates the coagulation cascade and release of inflammatory mediators, resulting in deposition of platelet and fibrin microthrombi, fibrocellular intimal proliferation, and smooth muscle colonization [2]. These processes lead to diffuse narrowing of the pulmonary arteriolar system and increased vascular resistance, resulting in pulmonary hypertension [3].

PTTM typically manifests with progressive dyspnea, hypoxia, and pulmonary hypertension and is often misdiagnosed as idiopathic pulmonary hypertension [1]. It progresses rapidly to right-sided heart failure and sudden death within weeks, often before the underlying tumor becomes clinically apparent [1].

Antemortem diagnosis of PTTM is difficult and, to date, the condition has been described primarily at autopsy. However, recent reports have suggested that antemortem diagnosis can be made using a number of sources. This includes pulmonary microvascular cytology on samples drawn through a wedged pulmonary artery catheter, which has a reported sensitivity of 80–88% and a specificity of 82–94% [4]. A V/Q scan is reported to be useful in differentiating between thromboembolism and PTTM based on the location, size, and number of perfusion defects and may have been beneficial in this case [5]. CT scan and chest radiograph are typically nondiagnostic. Franquet et al., however, identified bilateral and diffuse well-defined opacities that appeared as longitudinal branching structures (tree-in-bud) on thin-section CT [6]. Subtle densities of a similar tree-in-bud appearance were noted on CTPA in this case on admission. Furthermore, FDG-PET images reportedly showed multifocal abnormal FDG uptake in both lung fields [7]. More invasive measures including CT-guided lung biopsy and transbronchial lung biopsy have also been used successfully, but the patient's condition often precludes their use [8].

Treatment for PTTM has had limited success and further studies are needed to assess the optimal therapeutic strategy. One patient was treated successfully with chemotherapy, corticosteroids, and anticoagulation [9]. It is suggested that chemotherapy removes malignant cells from the pulmonary circulation and thus reduces the stimulus for thrombus formation and fibrointimal proliferation [9].

In summary, PTTM poses difficult diagnostic and therapeutic issues and should be considered a cause of rapidly progressive pulmonary hypertension, particularly in patients with known cancer.
